# Transcriptomics and metabolomics reveal the primary and secondary metabolism changes in *Glycyrrhiza uralensis* with different forms of nitrogen utilization

**DOI:** 10.3389/fpls.2023.1229253

**Published:** 2023-11-02

**Authors:** Ying Chen, Yu Bai, ZhengRu Zhang, YuanFan Zhang, Yuan Jiang, ShangTao Wang, YanJun Wang, Zhirong Sun

**Affiliations:** School of Chinese Materia Medica, Beijing University of Chinese Medicine, Beijing, China

**Keywords:** *Glycyrrhiza uralensis*, nitrogen forms, transcriptomics, metabolomics, primary metabolism, bioactive constituents, flavonoids

## Abstract

The roots and rhizomes of *Glycyrrhiza uralensis* Fisch. represent the oldest and most frequently used herbal medicines in Eastern and Western countries. However, the quality of cultivated *G. uralensis* has not been adequate to meet the market demand, thereby exerting increased pressure on wild *G. uralensis* populations. Nitrogen, vital for plant growth, potentially influences the bioactive constituents of plants. Yet, more information is needed regarding the effect of different forms of nitrogen on *G. uralensis*. *G. uralensis* seedlings were exposed to a modified Hoagland nutrient solution (HNS), varying concentrations of nitrate (KNO_3_), or ammonium (NH_4_)_2_SO_4_. We subsequently obtained the roots of *G. uralensis* for physiology, transcriptomics, and metabolomics analyses. Our results indicated that medium-level ammonium nitrogen was more effective in promoting *G. uralensis* growth compared to nitrate nitrogen. However, low-level nitrate nitrogen distinctly accelerated the accumulation of flavonoid ingredients. Illumina sequencing of cDNA libraries prepared from four groups—treated independently with low/medium NH_4_^+^ or NO_3_^-^ identified 364, 96, 103, and 64 differentially expressed genes (DEGs) in each group. Our investigation revealed a general molecular and physiological metabolism stimulation under exclusive NH_4_^+^ or NO_3_^-^ conditions. This included nitrogen absorption and assimilation, glycolysis, Tricarboxylic acid (TCA) cycle, flavonoid, and triterpenoid metabolism. By creating and combining putative biosynthesis networks of nitrogen metabolism, flavonoids, and triterpenoids with related structural DEGs, we observed a positive correlation between the expression trend of DEGs and flavonoid accumulation. Notably, treatments with low-level NH_4_^+^ or medium-level NO_3_^-^ positively improved primary metabolism, including amino acids, TCA cycle, and glycolysis metabolism. Meanwhile, low-level NH_4_^+^ and NO_3_^-^ treatment positively regulated secondary metabolism, especially the biosynthesis of flavonoids in *G. uralensis*. Our study lays the foundation for a comprehensive analysis of molecular responses to varied nitrogen forms in *G. uralensis*, which should help understand the relationships between responsive genes and subsequent metabolic reactions. Furthermore, our results provide new insights into the fundamental mechanisms underlying the treatment of *G. uralensis* and other *Glycyrrhiza* plants with different nitrogen forms.

## 1 Introduction

*Glycyrrhiza uralensis* Fisch., also known as Chinese licorice, is a leguminous plant species and is considered one of the most economically important medicinal plants globally (Rizzato et al., 2017). The roots and rhizomes of *G. uralensis* have long been used as herbal medicine and a natural sweetener, and are frequently employed as flavoring additives in the food and tobacco industries (Mochida et al., 2017). Pharmacological and clinical studies have shown that *G. uralensis* has therapeutic utility in treating viral infections, inflammation, diabetes, asthma, and liver and lung problems (Adhikari et al., 2021; Zhong et al., 2022). It has been identified that the multiple positive bioactivities of *G. uralensis* are mainly correlated to triterpene saponins and flavonoids, among which glycyrrhizic acid and liquiritin, isoliquiritigenin and their glycoside derivatives are the principal active constituents (Wang et al., 2021; Zhong et al., 2022).

Due to the effective use of *G. uralensis* in medicine, food additives, and commercial products as well as the increasing market demand in recent years, there is a significant demand for cultivating *G. uralensis.* Especially wild *G. uralensis* is limited and challenging to obtain from the national second-class plant protection regions (Yin et al., 2020). *G. uralensis*, a medicinal herb native to Asia’s semi-arid regions like desert grassland, desert edge, and loess hills with sandy loam characteristics, has a nitrogen nutrition deficiency that promotes the accumulation of its secondary metabolites (Chen et al., 2017). However, farmers cultivating *G. uralensis* often apply excessive nitrogen fertilizer to pursue higher yields, neglecting the need for adequate accumulation of active ingredients. This approach may not be conducive to the future sustainable development of *G. uralensis* industry. The low quality of cultivated *G. uralensis* continues to put pressure on its wild populations (Chen et al., 2017). Recently, scholars have applied exogenous hormones such as abscisic acid to stimulate the accumulation of glycyrrhizic acid, liquiritin, and other flavonoids in *G. uralensis* (Xiang et al., 2015; Qiao et al., 2017). However, their different effects on the physiological growth of *G. uralensis* were not well studied and still cannot be applied to *G. uralensis* production. Moreover, it was reported that inoculation of endophytic fungi in *G. uralensis* could improve its active ingredient content, but this technique remains in the laboratory research stage and cannot yet be stably applied to *G. uralensis* cultivation (Mohamad et al., 2018; Liu et al., 2023). Therefore, it is essential to seek effective measures to regulate the quality of *G. uralensis* during its cultivation management.

Nitrogen (N) is a crucial macro-element for plant growth and development. Plants mainly utilize nitrate (NO_3_^-^) and ammonium (NH_4_^+^) as N forms in natural soil (Luo et al., 2013). Typically, as H^+^ is generated during NH_4_^+^ assimilation while OH^-^ is produced during NO_3_^-^ assimilation, NH_4_^+^ is more readily available than NO_3_^-^ for utilization by plants since less energy is required during its assimilation (Liu B. Y. et al., 2017). It is well known that the uptake and transport of NO_3_^-^ are primarily determined by nitrate transporters (NRT) and NO_3_^-^ can be further transported and reduced to NH_4_^+^ by the sequential action of nitrate reductase (NR) and nitrite reductase (NiR), and NH_4_^+^ is then assimilated to amino acids through the glutamine synthetase (GS)/glutamate synthetase (GOGAT) pathway (Zhao et al., 2021). Moreover, the nitrogen metabolism process not only synthesizes the nitrogen-containing compounds vital to all life but also provides precursors for many intermediate products and other compounds. These include flavonoids and phenols in plants, which derive from phenylalanine and are closely linked with amino acid metabolism (Giorgi et al., 2009; Gong et al., 2022). In *G. uralensis* cultivation, nitrogen fertilization is indispensable to achieve satisfactory yield, even though nutrient-poor soils promote the accumulation of secondary components (Chen et al., 2017). It has been reported that the ratio of NH_4_^+^/NO_3_^-^ affects the accumulation of metabolites in cultivated *G. uralensis*. For example, glycyrrhizic acid and flavonoid content reached optimum levels at NH_4_^+^/NO_3_^-^ ratios of 15:15 and 20:10, respectively (Yin et al., 2014). Nevertheless, the underlying physiological and molecular mechanisms of different N forms (NH_4_^+^ or NO_3_^-^) utilization in *G. uralensis* remain largely unknown. Therefore, investigating the regulatory mechanisms and genes involved in *G. uralensis*’ response to different nitrogen forms can provide a theoretical basis for nitrogen fertilizer application in its cultivation production.

Currently, changes in gene expression and metabolite concentrations in response to NH_4_^+^ and NO_3_^-^ have been thoroughly studied in several “omics” analyses, such as NH_4_^+^-sensitive species (like *Arabidopsis thaliana*) and high NH_4_^+^ tolerant species (like rice) (Li et al., 2021). Moreover, metabolites and genes involved in responses to salt stress and drought adaption in *G. uralensis* have been identified by transcriptomics and metabolomics analyses (Wang et al., 2021; Li et al., 2023). However, little is known about the integrated metabolites and gene changes in *G. uralensis* under different nitrogen forms supplied. In the present study, we measured the growth biomass and main active constituents’ content of *G. uralensis* under varied nitrogen forms. Furthermore, transcriptomic and metabolomics approaches were employed to explore the differences in primary and secondary metabolism, including interactions between metabolic pathways under various concentrations of either NO_3_^-^ or NH_4_^+^ as the N source in *G. uralensis*. Differentially expressed genes (DEGs) among the structural genes involved in nitrogen metabolism, triterpenoid and flavonoid biosynthesis were identified using bioinformatics analysis, and putative biosynthesis networks were created. This work will not only facilitate the elucidation of predominant primary and secondary metabolism pathways in *G. uralensis* under different nitrogen forms at both the metabolic and gene levels, but it will also contribute to the further development of high-quality cultivated *G. uralensis*.

## 2 Materials and methods

### 2.1 Plant materials and treatment

*G. uralensis* seeds were obtained from China National Traditional Chinese Medicine Co., Ltd. These seeds were sown in pots and cultivated in a phytotron at a phytotron of Beijing University of Chinese Medicine, Beijing, China. On May 15th, *G. uralensis* seeds were sown in plastic pots of uniform size (upper diameter 16 cm, lower diameter 13 cm, and depth 17.5 cm), each filled with 2.6 kg sandy soil containing 0.312% organic matter, 0.035% N, 0.025% P and 1.62% K. Seeds were grown at photoperiod 12 h, temperature 25 ± 1°C, humidity 60 ± 5%, dark period 12 h, temperature 20 ± 1°C, humidity 40 ± 5%. After the emergence of two leaves, the plants were irrigated. All plants were watered with 400 mL of modified Hoagland nutrient solution (0.44 g/L CaCl_2_, 0.493 g/L MgSO_4_·7H_2_O, 0.136 g/L KH_2_PO_4_, 2.86 mg/L H_3_BO_3_, 1.81 mg/L MnCl_2_·4H_2_O, 0.22 mg/L ZnSO_4_·7H_2_O, 0.08 mg/L CuSO_4_·5H_2_O, 0.03 mg/L Na_2_MoO_4_·2H_2_O, 5.56 mg/L FeSO_4_·7H_2_O, 7.46 mg/L EDTA, PH=7.4) every 14 days, and cultivated at photoperiod 14 h with temperature 25 ± 1°C and humidity 60 ± 5%, dark period 10 h with temperature 20 ± 1°C and humidity 40 ± 5%. 1 mmol/L ammonium (NH_4_)_2_SO_4_, 3 mmol/L ammonium (NH_4_)_2_SO_4_, 2 mmol/L nitrate (KNO_3_) and 6 mmol/L nitrate (KNO_3_) were separately dissolved in modified Hoagland nutrient solution. On June 15th, the uniformly growing *G. uralensis* seedlings were randomly assigned to different nitrogen nutrient solutions, and presented as 1 mmol/L (NH_4_)_2_SO_4_ (low ammonium) treatment group (RTN), 3 mmol/L (NH_4_)_2_SO_4_ (medium ammonium) treatment group (RTH), 2 mmol/L KNO_3_ (low nitrate) treatment group (RTD), 6 mmol/L KNO_3_ (medium nitrate) treatment group (RTG) and modified HNS treatment group (Control, RTC). On September 17th, the roots of *G. uralensis* seedlings were biologically repeated sampling three times for each group, immediately frozen in liquid nitrogen, and stored at -80°C for RNA and metabolites extraction. And representative plants from each group were also selected to measure biomass.

### 2.2 Measurement of root biomass

In each group, the root length and diameter of representative plants were measured. Sample roots were dried at 60°C until a constant weight was achieved, after which the dry weight was determined.

### 2.3 Determination of main active ingredients content

Samples were freeze-dried and ground to a fine powder (30 Hz, 1.5 min) using a mixer mill (MM 400, Retsch). For extraction, sample powder (0.1 g) was mixed with 10 mL of 70% ethanol and subjected to ultrasonication for 40 min at 40°C before being centrifuged for 10 min at 6000 rpm. The supernatant solution was then filtered using a 0.22 μm syringe filter and transferred to vials for analysis. The content of the main active ingredients (glycyrrhizic acid, liquiritin, liquiritigenin, isoliquiritigenin, and kaempferol) of samples were analyzed using a Shimadzu LC-20AT HPLC system. An Agilent 5 TC-C_18_ column (4.6 mm×250 mm, 5 μm) was utilized for chromatographic separation. The mobile phase comprised acetonitrile (A) and 0.5% formic acid in water (B) followed at 1 mL/min in a gradient elution (0~6 min, 20%~30%A;6~10 min, 30%~42% A;10~20 min, 42%~60% A;20%~36 min, 60%~80% A;36~40 min, 80%~80% A;40~48 min, 80%~90% A;48~51min, 90%~90%). The column temperature was maintained at 30°C. An injection volume of 20 μL was used for each sample. The detection wavelengths were set at 280 nm for glycyrrhizic acid, liquiritin, liquiritigenin, and kaempferol, and 350 nm for isoliquiritigenin.

### 2.4 RNA extraction, Illumina library construction and sequencing

The total RNA of the samples was extracted with an RNAprep Pure Plant Kit following the manufacturer’s protocol (DP432, Tiangen, China). The samples harvested in triplication included the root of *G. uralensis* treated with low, medium ammonium (RTN, RTH) or nitrate (RTD, RTG) and without nitrogen (RTC). The extracted RNA was electrophoresis on a 1% agarose gel and examined using a NanoPhotometer spectrophotometer (IMPLEN, Los Angeles, CA, USA). A Qubit RNA Assay Kit and a Qubit 2.0 Fluorometer (Life Technologies, Carlsbad, CA, USA) were used to quantify RNA. Furthermore, RNA integrity was assessed using an RNA Nano 6000 Assay Kit and the Agilent Bioanalyzer 5400 system (Agilent Technologies, Santa Clara, CA, USA). The construction of Illumina sequencing libraries was carried out as previously described (Pertea et al., 2015). After amplification and purification, cDNA libraries were sequenced using the Illumina HiSeq platform (Illumina Inc., San Diego, CA, USA) by Metware Biotechnology Co., Ltd. (Wuhan, China).

### 2.5 Transcriptome data analysis

To obtain high-quality transcriptome data, adapters of sequences were cut, and low-quality reads with ≥5 uncertain bases or with over 50% Qphred ≤ 20 bases were removed using fastp. The GC content of clean reads was calculated. Fast QC also produced the Q20 and Q30 values to evaluate the base quality. Then, the clean reads were mapped to the *G. uralensis* reference genome using HISAT2 with default parameters. Gene function annotation was compared with the Kyoto Encyclopedia of genes and genomes (KEGG), non-redundant (NR), Swiss-Prot, euKaryotic Ortholog Groups (KOG), Pfam, gene ontology (GO) databases using BLAST software. Afterwards, gene expression levels were determined using the FPKM (fragments per kilobase of transcript per million mapped reads) method.

The read count was normalized by featiureCounts/StringTie. Moreover, the DEGs of treated plants relative to control samples were identified with the criteria |Log_2_Fold Change|≥1 and false discovery rate (FDR) < 0.05 using the DESeq2. Spearman’s correlation coefficient, GO enrichment analysis, and hierarchical cluster were performed and visualized by the libraries in the R language. Orthogonal partial least squares discriminant analysis (OPLS-DA) of expressed genes among all groups was conducted by SIMCA 14.1. The GO enrichment and KEGG pathway enrichment of DEGs were analyzed using the topGO method based on the hypergeometric distribution and KOBAS 3.0, respectively.

### 2.6 Metabolome analysis

Metabolite extraction and analysis followed previously described protocols (Chen et al., 2013). 100 mg of each freeze-dried sample powder was extracted using 1.2 mL 70% methanol solution and then was vortexed 30 s every 30 minutes six times and kept at 4°C overnight. Following centrifugation at 12000 rpm for 10 min, the extracts were filtrated with a 0.22 μm pore size membrane and stored in chromatographic sample bottles.

Then, the sample extracts were analyzed in positive and negative ionization mode on a UPLC system (SHIMADZU Nexera X2) equipped with an Applied Biosystems 4500 QTRAP MS and an Agilent UPLC SB-C_18_ (1.8 µm, 2.1 mm ×100 mm) column under the following conditions: mobile phase consisting of pure water with 0.1% formic acid (solvent A) and acetonitrile with 0.1% formic acid (solvent B), gradient program: 5% B (0 min), 5%-95% B (0-9 min), 95% B (9-10 min), 95%-5% B (10-11 min), 5%-5%B, (11-25 min), flow rate: 0.35 mL/min, the column oven temperature: 40°C, injection volume: 4 μL. LIT and triple quadrupole (QQQ) scans were acquired on a triple quadrupole-linear ion trap mass spectrometer (Q TRAP) equipped with an ESI Turbo Ion-Spray interface on an AB Sciex QTRAP 4500 System and controlled by Analyst 1.6.1 software. The ESI source operation parameters were as described previously.

Mass spectrometric data were processed with Analyst 1.6.3 software. Then, the quality and quantity of metabolites were processed with MultiaQuant based on a local metabolite database of Metware Biotechnology. For quality control (QC) analysis, one quality control sample was inserted into each of the ten tests and analysis samples to monitor the repeatability of the analytic process. Metabolite data were log_2_-transformed for statistical analysis to improve normality and then were normalized. The variable importance of the projection (VIP) score of the application OPLS-DA model was used to filter the best-differentiated metabolites between treatments. Metabolites with significant differences in content were set with thresholds of VIP≥1 and fold change≥2 or ≤0.5. Differentially accumulated metabolites (DAMs) were functional annotated based on KEGG pathways.

### 2.7 Co-joint analysis of the transcriptome and metabolome

The transcriptome and metabolome data were log-transformed (log_2_) for integration analysis. The differential genes and metabolites were mapped onto the KEGG pathways at the same time. The enrichment results of the differential genes and metabolites were used to show the degree of pathway enrichment. To study the correlations between the genes and metabolites, the Corson program in *R* was used to calculate Pearson’s correlation coefficients, which were presented as the nine quadrants.

### 2.8 qRT-PCR verification

The transcriptome results were verified by qRT-PCR. A total of 10 DEGs related to nitrogen metabolism were selected, namely *GuNRTs* (Glyur000276s00017180), *GuAMTs* (Glyur000347s00029040), *GuNR* (Glyur003792s00046197), *GuNiR* (Glyur000006s00001623), *GuGS* (Glyur000722s00018853), *GuGOGAT* (Glyur000616s00026610), *GuGDH* (Glyur000516s00022247), *GuGABA-T* (Glyur000167s00012262), *GuGAD* (Glyur000545s00023412), *GuSSADH* (Glyur002418s00033274). Purified RNA (1 μg for each sample) was reverse transcribed to the first strand cDNA using a PrimeScript™ cDNA Reverse Transcription reagent Kit with gDNA Eraser (Takara RR047A) based on the manufacturer’s instructions. Quantitative real-time PCR (qRT-PCR) was performed on a CFX96 real-time PCR system (Bio-Rad) using a Taq Pro Universal SYBR qPCR Master Mix kit (Vazyme). The primers used for the qRT-PCR were listed in **Supplementary Table S1**. For each target gene, three biological and technical replications were performed. Relative transcript levels were calculated against the reference *Actin* according to the 2^-ΔΔCt^ method.

## 3 Results

### 3.1 The effects of different nitrogen sources on the biomass of *G. uralensis*

Different forms of nitrogen had varying impacts on the growth and biomass accumulation of *G. uralensis* (**Supplementary Figure S1**). Among the treatment groups, RTG exhibited the most considerable root length, which was 12.85% significantly higher than that of the control. After exposure to various nitrogen treatments, there was an evident increase in the root diameter and dry weight of *G. uralensis*. Notably, RTH showed a significant enhancement in both root diameter and dry weight. Compared to the control, the root diameter and dry weight of RTH increased by 50.90% and 158.10%, respectively.

### 3.2 The effects of different nitrogen forms on the main active ingredients content of *G. uralensis*

The presence of ammonium or nitrate nitrogen variably affected the main active ingredient content of *G. uralensis* (**Supplementary Figure S2**). RTH treatment led to the highest accumulation of glycyrrhizic acid, followed by RTG. The glycyrrhizic acid content in RTH was 1.67-fold higher than that of the control. The flavonoid compounds liquiritin, liquiritigenin, and isoliquiritigenin showed different trends compared to glycyrrhizic acid. The RTD treatment resulted in the highest liquiritin, liquiritigenin, and isoliquiritigenin content in the root, registering increases of 1.48-fold, 1.46-fold, and 1.21-fold compared to the control, respectively. Furthermore, RTC exhibited a notably higher kaempferol content.

### 3.3 Transcriptome profiling of *G. uralensis*

**Figure 1:**
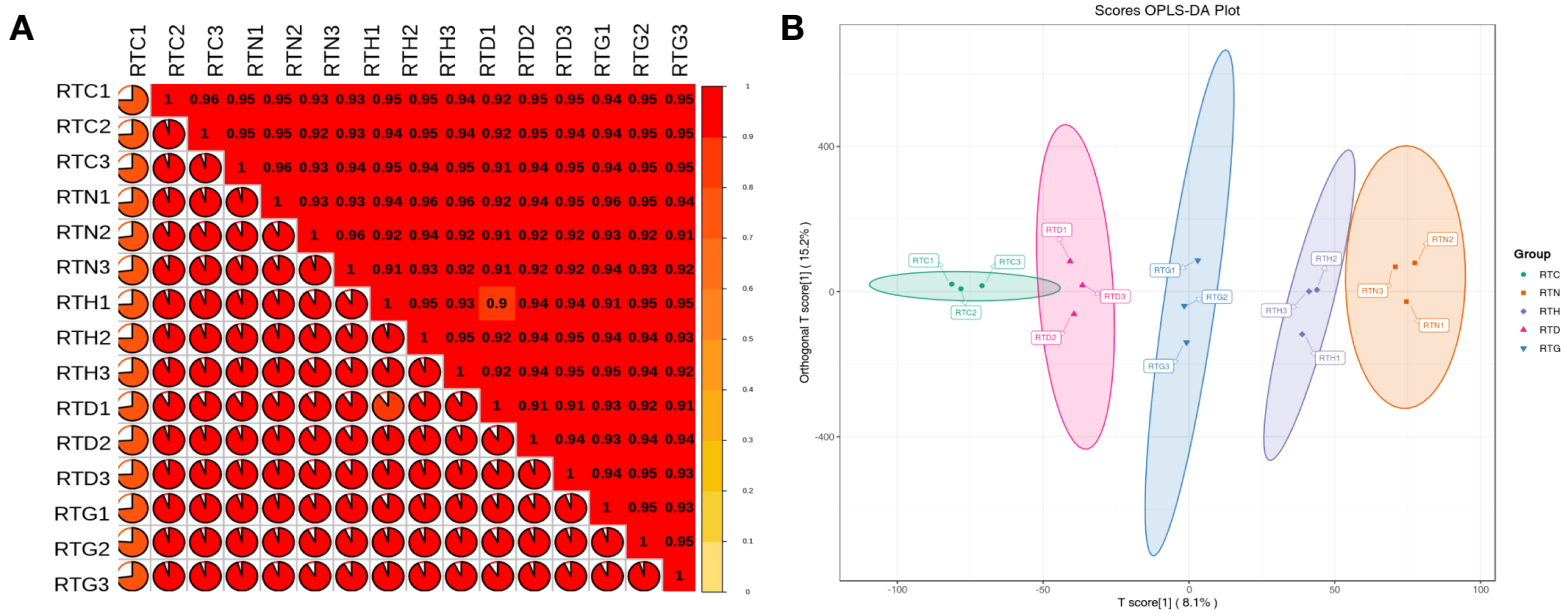
*G. uralensis* transcriptomic parameter analyses. **(A)** Pearson’s correlations of different forms of nitrogen treatment *G. uralensis* samples. **(B)** Orthogonal partial least squares discriminant analysis of expressed genes.

Illumina HiSeq 6000 paired-end sequencing yielded a total of 68.60 million and named RTC, RTN, RTH, RTD, and RTG. The Q30 percentage was > 92% for each library, and the average GC content was approximately 45% for all libraries, indicating that the quality of transcriptome sequencing data was high (**Table 1**). Among these, 71.99%-92.14% of clean reads were mapped to the reference genome of samples. Based on Pearson’s correlation analysis between samples, it can be observed that there were reliable biological replicates within the group between samples ranging from 0.92-0.95 (**Figure 1A**). The OPLS-DA showed all groups were well separated, suggesting significant differences existed in gene expression among groups (**Figure 1B**).

**Table 1:** Summary of the sequencing data from the 15 *G. uralensis* samples.

Sample	Clean Reads	Clean Base(G)	Q20/%	Q30/%	GC Content/%	Reads mapped/%
RTC1	43232966	6.48	97.45	92.72	44.97	92.14
RTC2	46238408	6.94	97.58	93.1	45.04	91.76
RTC3	41747658	6.26	97.41	92.6	44.7	91.52
RTN1	42379410	6.36	97.38	92.64	44.99	91.82
RTN2	51042782	7.66	97.34	92.54	46.07	71.99
RTN3	44571494	6.69	97.35	92.58	45.27	78.11
RTH1	45618400	6.84	97.39	92.57	45.19	91.56
RTH2	45352252	6.8	97.39	92.66	45.3	90.98
RTH3	44065792	6.61	97.27	92.36	45.35	91.63
RTD1	43800622	6.57	97.32	92.46	44.71	91.98
RTD2	46296932	6.94	97.34	92.54	45.26	91.45
RTD3	42014346	6.3	97.25	92.42	45.61	1.07
RTG1	48081362	7.21	97.2	92.18	44.98	87.7
RTG2	56539276	8.48	97.49	92.84	45.31	90.61
RTG3	45040284	6.76	97.36	92.49	44.99	91.75

### 3.4 Differential gene expression analysis of *G. uralensis*

**Figure 2:**
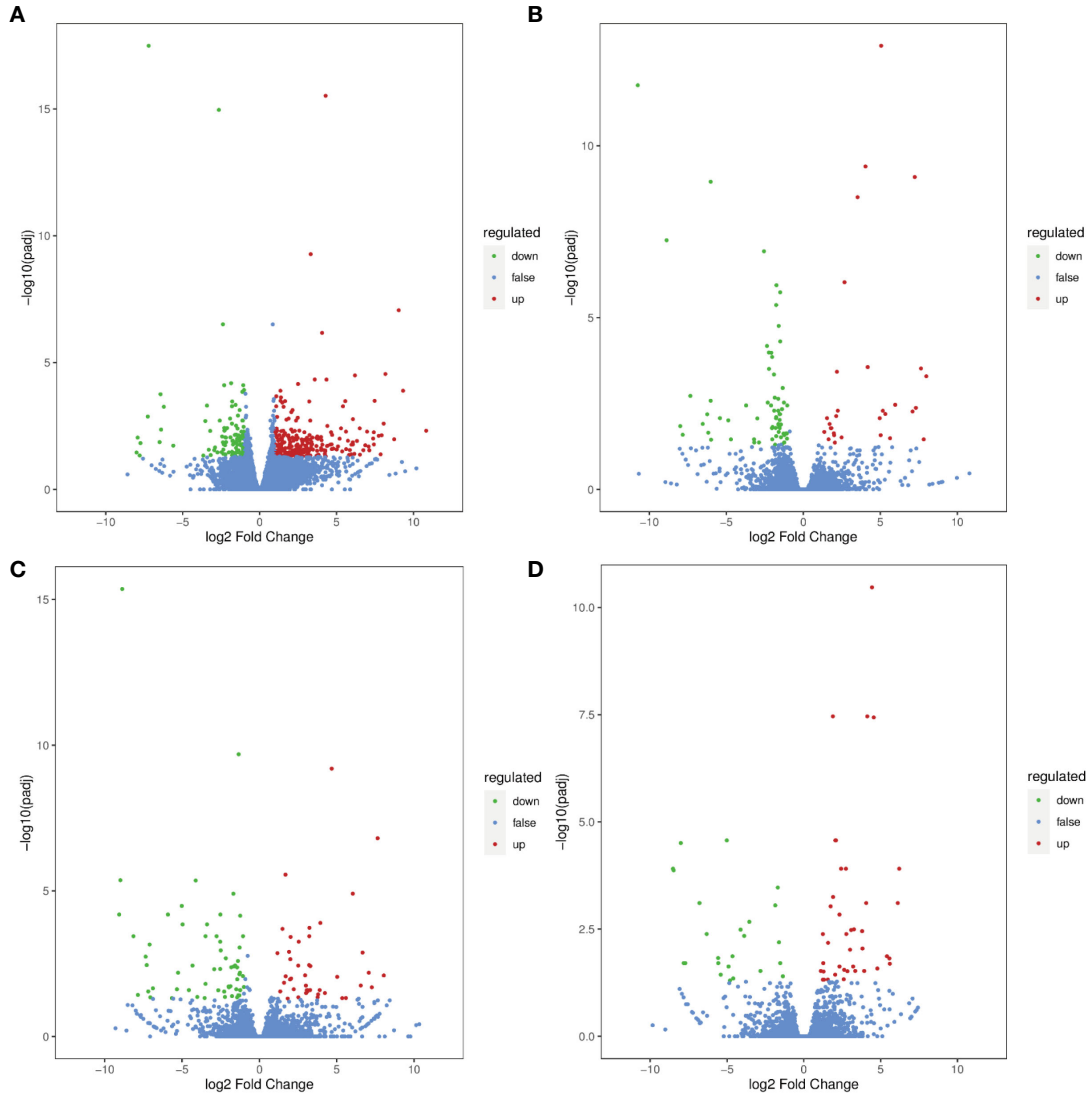
Differential gene volcano maps of different forms of nitrogen treatment *G. uralensis* samples. **(A)** RTC_vs_RTN, **(B)** RTC_vs_RTH, **(C)** RTC_vs_RTD, **(D)** RTC_vs_RTG. The significant DEGs with Log_2_ (fold change)| ≥1 and FDR < 0.05 are represented by green (down-regulated) and red (up-regulated) dots.

Volcano plots were constructed to determine the number of DEGs significantly changed in *G. uralensis* samples. Based on the threshold of |Log_2_Fold Change |≥1 and FDR< 0.05, 258, 30, 43, and 40 DEGs were upregulated, and 106, 66, 60 and 24 DEGs were downregulated in RTC_vs_RTN, RTC_vs_RTH, RTC_vs_RTD, and RTC_vs_RTG, respectively (**Figure 2**). The Venn diagram revealed that 5 DEGs were shared among four comparisons (**Supplementary Table S2**). Moreover, 328, 67, 77, and 45 DEGs were expressed explicitly in the RTC_vs_RTN, RTC_vs_RTH, RTC_vs_RTD, and RTC_vs_RTG, respectively (**Supplementary Figure S3**).

**Figure 3:**
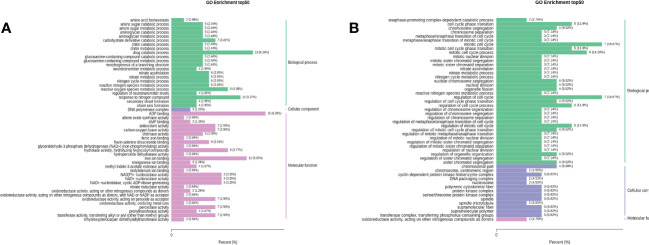
Gene ontology (GO) functional classifications of differentially expressed genes (DEGs) of RTC_vs_RTN **(A)** and RTC_vs_RTH **(B)**.

To further understand the biological function of the DEGs, GO analysis was performed to distribute the DEGs into three major functional categories: Cell Component (CC), Molecular Function (MF), and Biological Process (BP). The DEGs of RTC_vs_RTN, RTC_vs_RTH, RTC_vs_RTD, and RTC_vs_RTG were significantly enriched in 1320, 464, 527, and 319 GO terms, respectively. The top 50 GO terms of RTC_vs_RTN, RTC_vs_RTH were presented in **Figure 3**. The most enriched components of RTC_vs_RTN in the BP were the drug catabolic process (GO:0042737) and response to nitrogen compound (GO:1901698), whereas ADP binding (GO:0043531) was the most enriched GO term in the MF (**Figure 3A**). GO term enrichment of RTC_vs_RTH was shown in **Figure 3B**, where the most genes enriched were those related to the regulation of cell cycle (GO:0051726) and mitotic cell cycle (GO:0000278) in the BP category, and chromosomal part (GO:0044427) was the most enriched GO term in CC. The GO terms of RTC_vs_RTD and RTC_vs_RTG were different (**Supplementary Figure S4**). The mainly highly enriched term in RTC_vs_RTD was a response to ion homeostasis (GO:0050801), cation homeostasis (GO:0055080) and cellular chemical homeostasis (GO:0055082) in BP, while the most enriched GO term of RTC_vs_RTG in BP was nitrogen cycle metabolic process (GO:0071941). Moreover, other terms related to nitrogen metabolism, including nitrate metabolic process (GO:0042126), nitrate assimilation (GO:0042128), nitrogen cycle metabolic process and reactive nitrogen species metabolic process (GO:2001057) were highly represented terms in the four pairwise comparisons.

**Figure 4:**
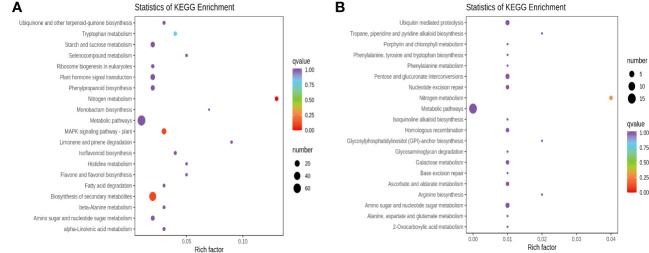
DEGs KEGG enrichment of top 20 pathways in RTC_vs_RTN **(A)** and RTC_vs_RTH **(B)**.

DEGs were further assigned to the KEGG database after screening for pathways with a value of *P* < 0.05, and the top 20 categories enriched in the KEGG pathways were presented. Apart from nitrogen metabolism (ko00910), MAPK signaling pathway-plant (ko04016), biosynthesis of secondary metabolites (ko01110), tryptophan metabolism (ko00380), and phenylpropanoid biosynthesis (ko00940) were the top five significantly enriched pathways for RTC_vs_RTN (**Figure 4A**), and the other pathways were primarily related to isoflavonoid biosynthesis (ko00943), flavone and flavonol biosynthesis (ko00944). For the RTC_vs_RTH (**Figure 4B**), the top five most enriched pathways were nitrogen metabolism, ubiquitin-mediated proteolysis (ko04120), nucleotide excision repair (ko03420), pentose and glucuronate interconversions (ko00040) and amino sugar and nucleotide sugar metabolism (ko00520). In addition, the KEGG annotation showed that the most enriched pathways of RTC_vs_RTD were nitrogen metabolism, mismatch repair (ko03430), and pyrimidine metabolism (ko00240) (**Supplementary Figure S5A**). Nitrogen metabolism, arginine and proline metabolism (ko00330), arginine biosynthesis (ko00220), and sesquiterpenoid and triterpenoid biosynthesis (ko00909) were highly enriched pathways in RTC_vs_RTG (**Supplementary Figure S5B**). Notably, metabolic pathways (ko01100) were enriched in RTC_vs_RTD and RTC_vs_RTG.

### 3.5 Differentially expressed transcription factors of *G. uralensis* under different nitrogen treatment

A total of 26 transcription factors (TFs) were differentially expressed in RTC_vs_RTN (**Supplementary Table S3**). These TFs were divided into 18 families. The major TFs identified in this study included CRAS (4), WRKY (3), AP2/ERF (2), TCP (2), C2H2 (2), MYB (1), bHLH (1), bZIP(1). Most TFs were up-regulated among these families, except 1 AP2/ERFs, 1 C2C2-GATAs, and 1 LIMs were down-regulated, respectively.

In RTC_vs_RTH, 2 TFs were differentially expressed and up-regulated, which belong to GRAS and LOB families, respectively. In RTC_vs_RTD, 3 TFs were differentially expressed and divided into three families, including AP2/ERF (1), LIM (1), and HB-KNOX (1). 1 AP2/ERF and 1 LIM were down-regulated, while 1 HB-KNOX was up-regulated. In RTC_vs_RTG, 2 TFs were differentially expressed, including MYB (1) and LOB (1), which were up-regulated. Most of the TFs were up-regulated in *G. uralensis* after different nitrogen treatments, indicating that these TFs mainly responded to the various nitrogen forms treatment through positive feedback regulation.

### 3.6 Metabolomics profiles of *G. uralensis* supplied under different nitrogen sources

Widely targeted metabolome was subsequently performed using an LC-ESI-MS/MS system to identify the metabolites related to *G. uralensis* under different nitrogen sources supplements. And a total of 996 metabolites from all samples were found, including amino acids and derivatives, organic acids, terpenoids, flavonoids, phenolic acids, lignans, coumarins, alkaloids and other substances. The OPLS-DA analysis showed that different forms of nitrogen treatment groups were separated clearly from control groups with fewer variables within groups, indicating that the metabolomics data had high reliability and reproducibility (**Supplementary Figure S6**).

After combining the results of VIP≥1.0 and fold change≥2 or ≤0.5 as thresholds for significant differences, a total of 113 DAMs were identified in RTC_vs_RTN, among which 79 were upregulated, and 34 were downregulated, including 6 alkaloids, 6 amino acids and derivatives, 29 flavonoids, 2 lignans and coumarins, 2 organic acids, 11 phenolic acids, 9 terpenoids and 48 other metabolites (**Supplementary Table S4**). In RTC_vs_RTH, the following DAMs (64 up- and 50 down-regulated) were identified: 1 alkaloid, 6 amino acids and derivatives, 35 flavonoids, 1 lignan and coumarin, 4 organic acids, 12 phenolic acids, 5 terpenoids and 50 other metabolites (**Supplementary Table S5**). The following DAMs (32 up- and 52 down-regulated) were identified in RTC_vs_RTD comparison: 2 alkaloids, 10 amino acids and derivatives, 28 flavonoids, 3 lignans and coumarins, 2 organic acids, 10 phenolic acids, 4 terpenoids and 50 other metabolites (**Supplementary Table S6**). Besides, 171 DAMs (60 up- and 111 down-regulated) were identified in RTC_vs_RTG containing 4 alkaloids, 5 amino acids and derivatives, 56 flavonoids, 6 lignans and coumarins, 9 organic acids, 28 phenolic acids, 8 terpenoids, and 55 other metabolites (**Supplementary Table S7**).

**Figure 5:**
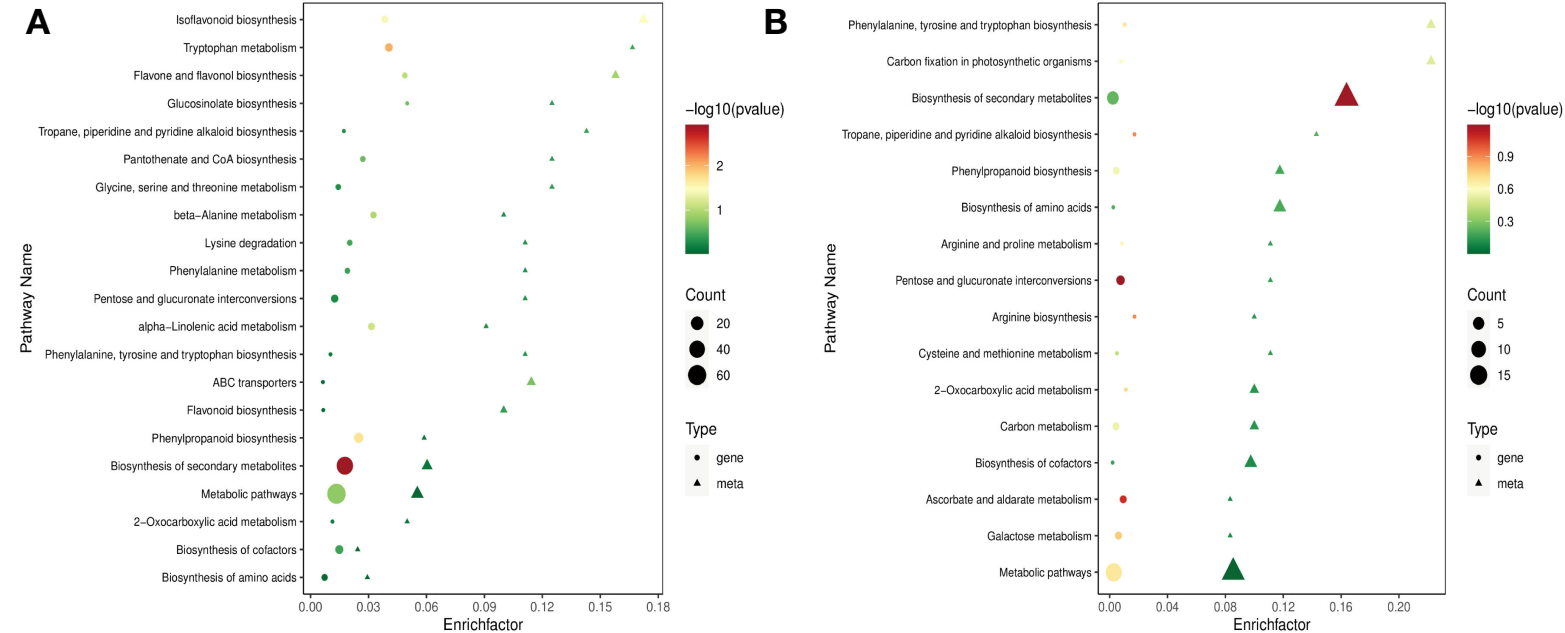
Joint pathways of DEGs and DAMs KEGG enrichment in RTC_vs_RTN **(A)** and RTC_vs_RTH **(B)**.

The co-joint pathways of DEGs and DAMs KEGG enrichment analysis of sample groups were conducted. The results showed that 47 of 113 DAMs were assigned 21 pathways in RTC_vs_RTN (**Figure 5A**). These DAMs were the main components for the isoflavonoid biosynthesis (ko00943), flavone and flavonol biosynthesis (ko00944), biosynthesis of secondary metabolites (ko01110), phenylpropanoid biosynthesis (ko00940) and flavonoid biosynthesis (ko00941). In RTC_vs_RTH, 61 of 114 DAMs were enriched in 16 metabolic pathways. The most enriched pathways were the biosynthesis of secondary metabolites, phenylpropanoid biosynthesis, and biosynthesis of amino acids (ko01230) (**Figure 5B**). Among 84 DAMs identified in RTC_vs_RTD, 49 metabolites were allocated to 16 metabolic pathways, which were most enriched in nitrogen metabolism (ko00910), biosynthesis of secondary metabolites, and isoflavonoid biosynthesis (**Supplementary Figure S7A**). In addition, 58 of 171 DAMs were allocated to 10 metabolic pathways in RTC_vs_RTG. The significantly enriched pathways were phenylalanine metabolism (ko00360), biosynthesis of secondary metabolites, and biosynthesis of amino acids (**Supplementary Figure S7B**). Therefore, different nitrogen forms significantly influenced the biosynthesis of secondary metabolites and amino acids in *G. uralensis*. Notably, pathways in nitrogen metabolism, phenylpropanoid biosynthesis, and flavonoid biosynthesis were also enriched in *G. uralensis* samples. These data indicated that nitrogen forms, whether NH_4_^+^ or NO_3_^-^, affected the amino acid and sugar metabolism in *G. uralensis*.

To elucidate the correlation between the transcriptome and metabolome of *G. uralensis* with the supplement of different nitrogen sources, Pearson’s correlation coefficients of *r* > 0.8 or *r* < -0.8 were further constructed as nine quadrants (**Supplementary Figure S8**). The gene and metabolite differential expression patterns were consistent in both the third and seventh quadrants, in which genes were positively related to metabolite regulation, indicating that genes may positively regulate metabolite changes.

### 3.7 Integrated analysis of genes and metabolites involved in the nitrogen metabolism and amino acid synthesis

**Figure 6:**
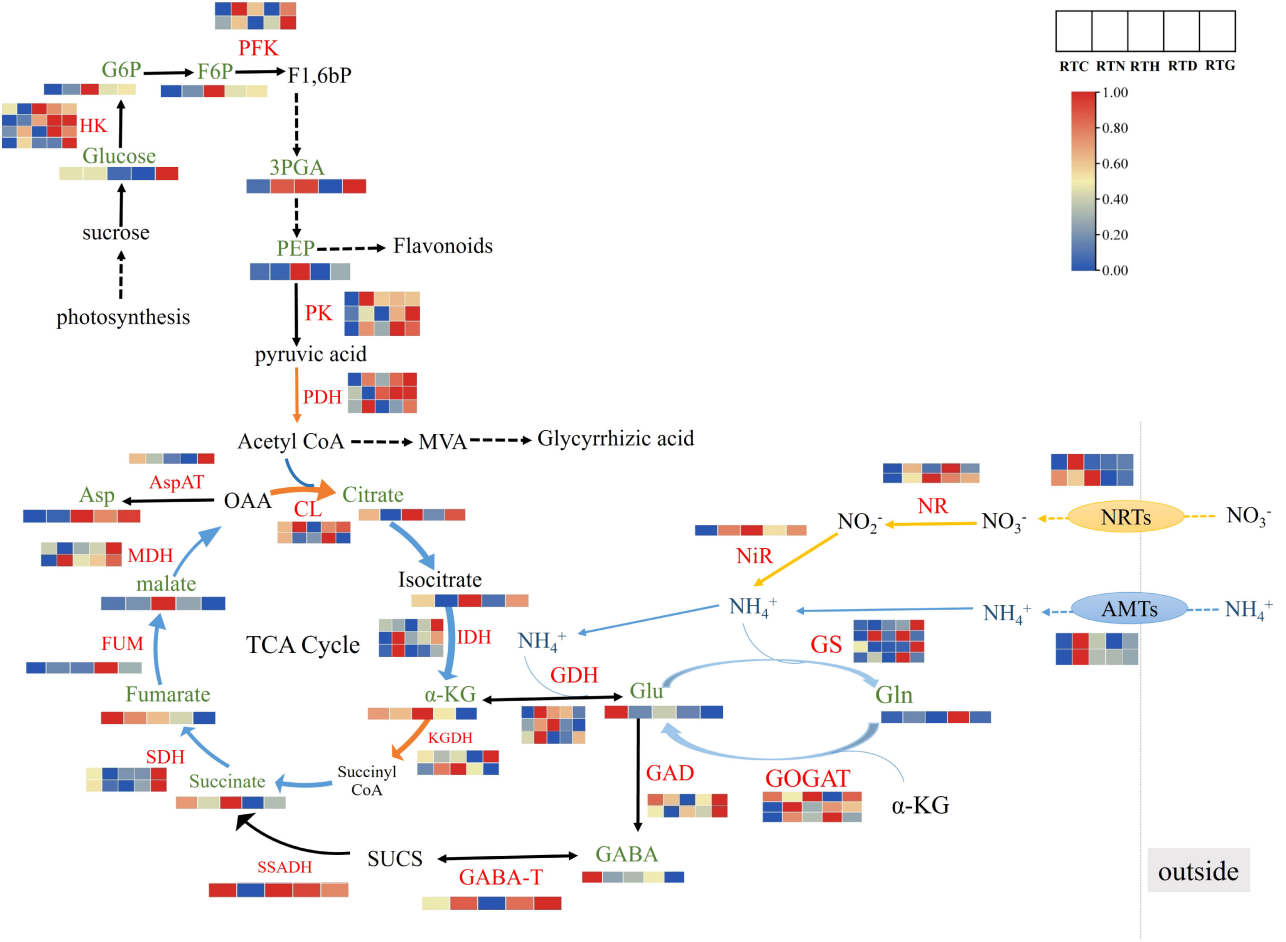
The DEGs and DAMs involved in nitrogen metabolism and amino acid synthesis pathways in *G. uralensis* response to different nitrogen forms treatment.

Based on the results obtained by gene and metabolic profiling, the effects of different nitrogen sources on relevant metabolic and their corresponding transcript levels in nitrogen metabolism and amino acid synthesis pathways were further studied (**Figure 6**). In plants, primary N metabolism including nitrate reduction and subsequent N assimilation are essential to plant growth and development. NR and NiR could catalyze the reduction from nitrate to ammonium, which is then converted into glutamate and glutamine through GS, GOGAT, and GDH (Arnao et al., 2022). The levels of gene expression related to NRTs and ammonium transporter (AMTs) were up-regulated in RTN, while genes encoding NR and NiR both high expressed in RTH. Specifically, the genes in GS/GOGAT pathway were up-regulated in RTN and RTD, and the GDH gene was up-regulated in RTN and RTH, the corresponding product glutamine (Gln) was up-regulated in RTD, whereas the content of glutamate (Glu) in the control group was higher than other N treatment groups. In plants, the GS/GOGAT pathway is considered the main pathway of ammonia assimilation. Nitrogen assimilation required a source of *α*-ketoglutarate (*α*-KG), which played a key role as the primary C-acceptor in the GS/GOGAT pathway for glutamate family amino acid synthesis (Hodges, 2002). The intermediates in glycolysis and the TCA cycle could be used as precursors for amino acid synthesis (Zhang et al., 2017). Conventionally, the TCA cycle begins with synthesizing citrate using oxaloacetate (OAA) and acetyl-CoA as substrates, proceeds via a series of oxidative reactions and ends with the regeneration of oxaloacetate. In this study, the content of *α*-KG and succinate were up-regulated along with the gene expression of KGDH in RTH in the TCA cycle. In contrast, citrate, isocitrate, and malate content in RTN were down-regulated, which was inconsistent with the gene expression of CL and IDH. In addition, *γ*-Aminobutyric acid (GABA) is metabolized via a short pathway known as the GABA shunt, which bypasses two steps of the TCA cycle as one of the bridges between N and C metabolism (Michaeli and Fromm, 2015). GABA is mainly produced by the irreversible of the cytosolic enzyme GAD, and GABA catabolism by the action of GABA-T to produce succinic semi-aldehyde (SUCS), then SUCS is converted by the enzyme SSA dehydrogenase (SSADH) to succinate that acts as a component of the TCA (Liao et al., 2021). Notably, gene expression levels of GAD and SSADH were enhanced in RTC, consistent with the changes of related metabolites like GABA and succinate. Meanwhile, the gene expression of GAD and GABA-T were up-regulated in RTG. Pyruvate was an essential metabolite that linked glycolysis and the TCA cycle together, which was synthesized via the catalysis of PK and then irreversibly converted into acetyl-CoA via PDH (Nie et al., 2020). Three essential enzyme genes, including PFK, PK, and PDH were up-regulated in RTN and RTG, in which the downstream compound 3PGA was also improved. Furthermore, the contents of G6P, F6P, 3PGA, and PEP were all enhanced in RTH compared to the control group.

### 3.8 Integrated analysis of genes and metabolites related to the flavonoid biosynthesis of *G. uralensis* under different nitrogen forms

**Figure 7:**
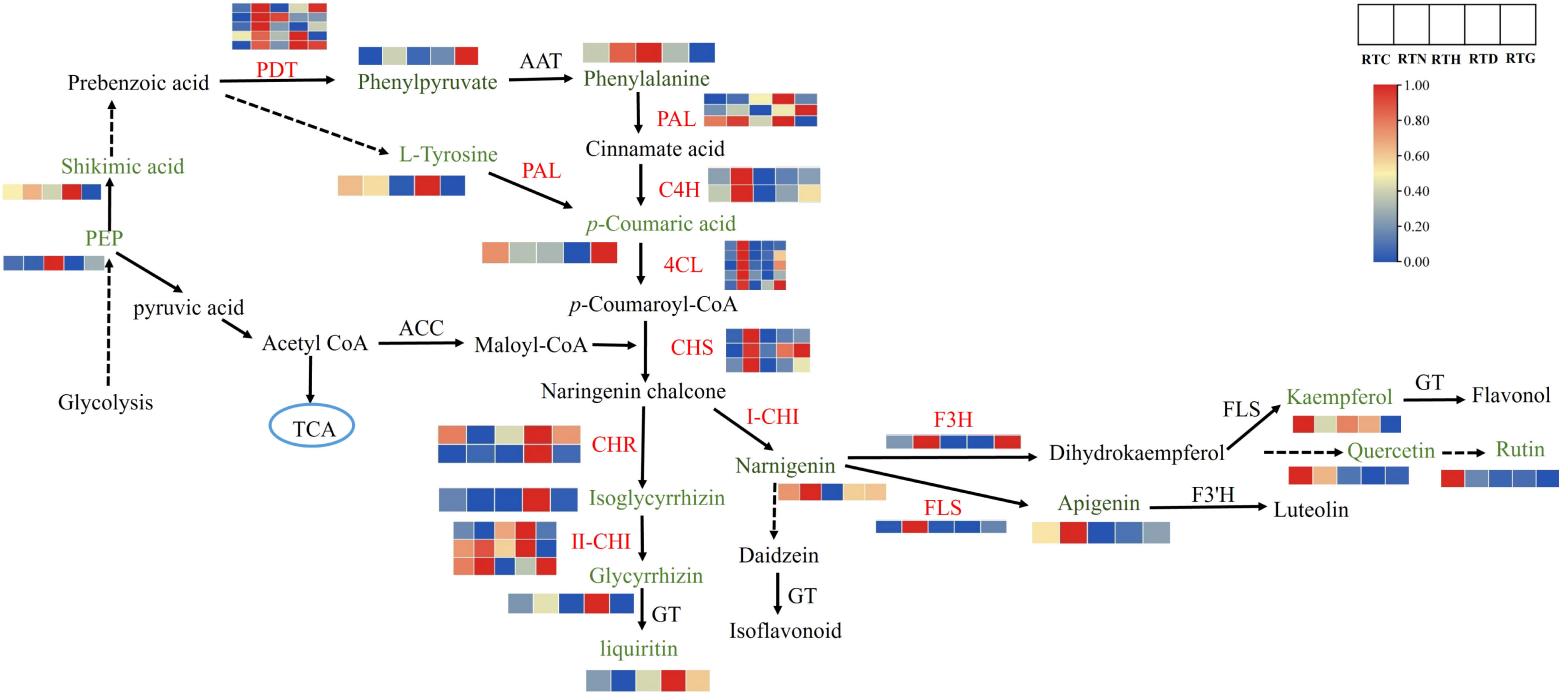
The DEGs and DAMs involved in flavonoid biosynthesis in *G. uralensis* response to different nitrogen forms treatment.

The transcriptome and metabolome analysis revealed that flavonoid biosynthesis played a vital role in the *G. uralensis* in response to different nitrogen forms. Therefore, a putative network of flavonoid biosynthesis was assigned based on the enriched KEGG pathways and gene functional annotation (**Figure 7**). Flavonoid biosynthesis originates from the phenylpropanoid pathway (Zhang et al., 2021). The three enzymes of PAL, C4H, and 4CL are the key enzymes at the beginning of the phenylpropanoid pathway, catalyzing the formation of cinnamate acid and *p*-coumaroyl-CoA, which is a substrate for the synthesis of various flavonoid constituents (Docimo et al., 2013). Three PALs were obtained, including Glyur000055s00004545, Glyur000226s00010834, and Glyur000249s00015874, and three genes were various up-regulated in RTN, RTD, and RTG compared to the control group. Two putative C4Hs (Glyur001446s00035038 and novel.5053) and five 4CLs (Glyur000051s00003417, Glyur000098s00007989, Glyur000681s00027307, Glyur000731s00024682, Glyur000910s00020578) were identified in all groups, and these genes all presented higher expression levels in RTN than the control.

In the downstream of the pathway, the expression of DEGs also showed a noticeable difference in *G. uralensis* after different form nitrogen treatments. CHS is the first rate-limiting enzyme in the flavonoid biosynthesis pathway that mediates the isomerization of naringenin chalcone by catalyzing the condensation of *p*-coumaroyl-CoA and malonyl-CoA (Zang et al., 2019). It was found that the expression levels of three putative CHSs (Glyur000051s00003428, Glyur000336s00018105 and Glyur000397s00020478) increased by twofold or threefold in RTN contrast to control. Afterwards, two CHRs (Glyur000218s00011631 and Glyur000784s00031237) were identified that catalyze naringin chalcone to isoglycyrrhizin. Moreover, CHI, the critical enzyme catalyzing isoglycyrrhizin to glycyrrhizin, was also obtained. The expression levels of CHR and CHI were up-regulated in RTD and RTG. The glycyrrhizin content in RTD also increased with the expression of genes such as CHI. The flavonoid biosynthesis pathway then diverges into the side branch, producing the top product naringenin (Liu W. et al., 2017). Putative F3H (Glyur000140s00011488) and FLS (Glyur002747s00042594) can convert naringenin to dihydrokaempferol and kaempferol, respectively. The F3H gene was up-regulated in RTN and RTG. However, the downstream products kaempferol, quercetin, and rutin were downregulated in treated groups and not in accordance with the expression of the corresponding genes as F3H and FLS. In addition, FLS contributes to converting narnigenin to apigenin showed upregulated in RTN, and the content of apigenin showed enhanced expression levels of RTN in following the expression of the corresponding gene.

### 3.9 Integrated analysis of genes and metabolites related to the triterpenoid biosynthesis of *G. uralensis* under different nitrogen forms

**Figure 8:**
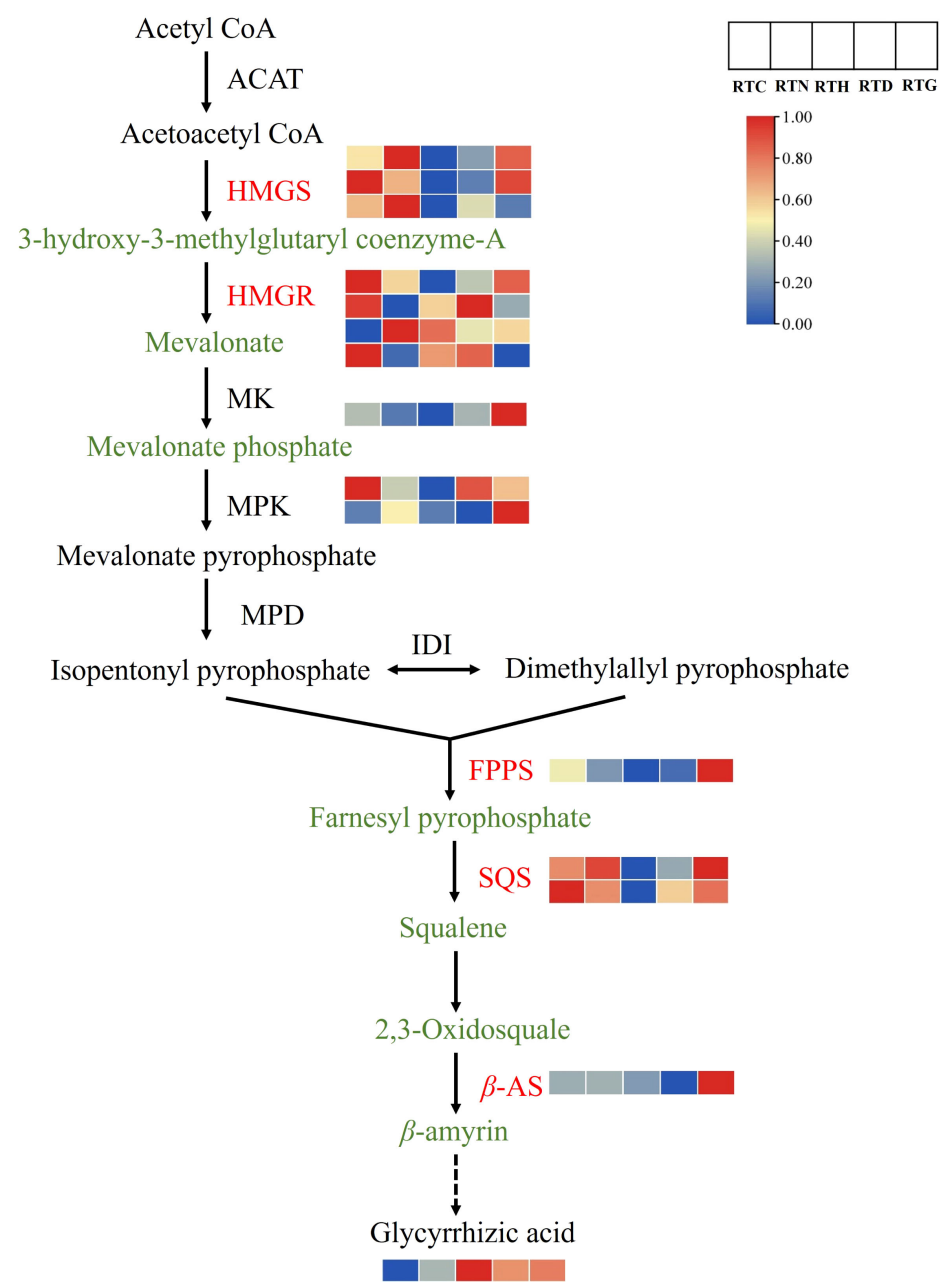
The DEGs and DAMs involved in triterpenoid biosynthesis in *G. uralensis* response to different nitrogen forms treatment.

Furthermore, a putative triterpenoid biosynthesis network revealed that treatments with different nitrogen forms influenced the precursor steps of glycyrrhizic acid biosynthesis (**Figure 8**). Glycyrrhizic acid synthesis follows the mevalonate (MVA) pathway, which begins with the primary metabolite, acetyl CoA, and encompasses three pivotal stages. Within the MVA pathway, HMGR stands out as the initial rate-limiting enzyme. The MVA is produced through the combined activities of ACAT, HMGS, and HMGR. Followed by isopentenyl pyrophosphate and dimethylallyl pyrophosphate synthesis, which are used as precursors under the action of MK, MPK, and MPD to form terpenoid metabolites. Notably, the HMGR gene displayed varied up-regulation across treatment groups, while MK and MPK gene expressions surged in RTG compared to the control group. Further downstream, a universal precursor for the biosynthesis of sterols and triterpenes, 2,3-Oxidosquare is produced under the collaborative actions of FPPS and SQS. Glycyrrhizic acid is then synthesized through a series of enzymatic catalysis with enzymes like *β*-AS. It is noteworthy that FPPS, SQS, and *β*-AS genes were up-regulated in RTG, and the downstream product glycyrrhizic acid content increased in RTN, RTD, and RTG compared to that of the control.

### 3.10 Confirmation of gene expression of nitrogen metabolism using qRT-PCR

**Figure 9:**
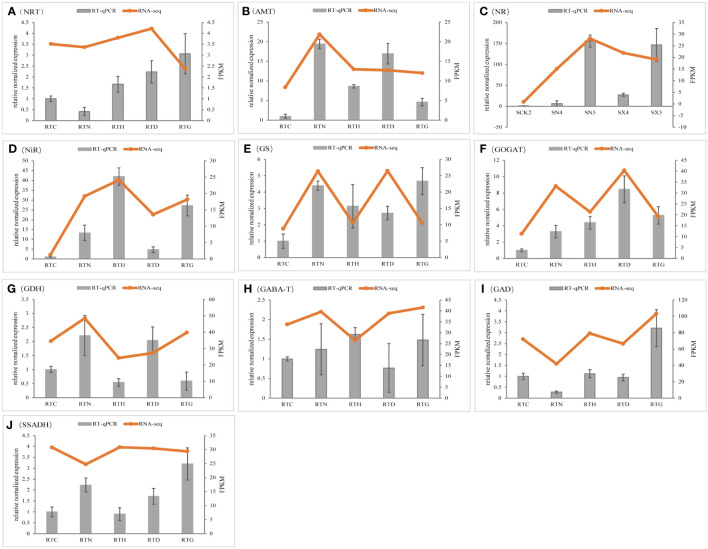
Validation of gene expression by qRT-PCR analysis. The data value is the average expression value of the transcriptome and qRT-PCR data. The error bar represents the Standard Error of the Mean. **(A)** NRT, **(B)** AMT,**(C)** NR, **(D)** NiR, **(E)** GS, **(F)** GOGAT, **(G)** GDH,**(H)** GABA-T, **(I)** GAD, **(J)** SSADH.

To validate the reliability of the results from RNA-Seq, further analysis of the expression of genes that might regulate nitrogen metabolism in *G. uralensis* was performed by qRT-PCR. The expression patterns of these genes assayed using RT-qPCR were broadly consistent with RNA-seq data, and data from the two methods had a high correlation coefficient (*R* ^2^ = 0.9567) (**Figure 9**).

## 4 Discussion

Nitrate and ammonium serve as the two primary inorganic nitrogen sources plants require (Li et al., 2016). However, they elicit distinct responses in plants’ developmental and physiological processes, including root morphology, N assimilation and metabolism (Oldroyd and Leyser, 2020). *G. uralensis*, a resilient plant species, thrives in low-fertility soils. Its natural distribution targets nutrient-poor soil environments. However, during its cultivation, intensive chemical fertilization is employed to achieve satisfactory yields, contributing to the deterioration of soil properties and environmental pollution (Chen et al., 2017). Our previous research indicated that *G. uralensis* rapidly accumulated both above-ground and below-ground biomass weight, as well as glycyrrhizic acid content, from early to late September in a dynamic sampling experiment (Liu W. L. et al., 2017). Therefore, this study simulated a natural low nitrogen environment, setting low and medium concentration nitrogen levels, and sampled in mid-September to observe the effects of different nitrogen forms on the growth and active ingredient content of *G. uralensis*. Here, we found that the impact of medium concentration ammonium nitrogen in promoting the root diameter and dry weight of *G. uralensis* was greater than that of nitrate nitrogen. This result may be because ammonium nitrogen is usually the preferred N source by plants due to its reduced equivalents and metabolic costs (Li et al., 2021). Previous studies suggest that appropriate environmental stress, such as nitrogen deficiency, would increase the accumulation of glycyrrhizin and several flavonoids in *G. uralensis* (Yin et al., 2014; Dong et al., 2021; Liu Y. et al., 2021). Our research showed the same accumulation trend of the main flavonoid ingredients in *G. uralensis* under low ammonium or nitrate nitrogen treatment. Low nitrate nitrogen treatment more greatly accelerated the accumulation of flavonoid compounds such as liquiritin, liquiritigenin, and isoliquiritigenin than other treatments. Additionally, low ammonium nitrogen improved the liquiritigenin content of *G. uralensis* compared to the control. Interestingly, the glycyrrhizic acid content increased with the proper concentration of either ammonium or nitrate nitrogen supplied. Nonetheless, the molecular mechanisms governing the biosynthesis of these constituents in *G. uralensis* under varying nitrogen supplies remain unclear. This knowledge gap significantly hampers the cultivation of high-quality *G. uralensis* as well as the broad and effective utilization of its resources. Thus, we further analyzed and compared the transcriptomics and metabolomics differences among the control groups and various concentrations of NH_4_^+^ or NO_3_^-^ treated cultivated *G. uralensis* groups, which aims to shed light on the *G. uralensis*’s response and adaptations to different nitrogen forms.

To delve deeper into the effects of different nitrogen forms on *G. uralensis* metabolism, we studied the expression levels of key enzymes involved in related metabolic pathways. We validated a subset of DEGs linked to responses unique to NH_4_^+^ or NO_3_^-^ supply by conducting GO and KEGG enrichment analyses. KEGG enrichment primarily served to scrutinize primary and secondary metabolite biosynthesis. Our metabolite analysis identified 996 chemicals, which included amino acids and derivatives, organic acids, terpenoids, flavonoids, phenolic acids, and other substances. Among these, flavonoids and triterpenoids constituted the highest chemical proportion and primarily contributed to the bioactive benefits of *G. uralensis*. The contents of amino acids underwent significant changes under NH_4_^+^ or NO_3_^-^ supplied in our samples. For instance, we noted downregulation of glutamate (1.29-fold) and aspartate (0.69-fold), and upregulation of glutamine (1.24-fold) in the RTN group. Additionally, aspartate showed 1.40-fold upregulation in the RTG group. These patterns closely mirrored the gene expression trends in our transcriptome. The GS/GOGAT pathway is recognized as the primary route of ammonia assimilation, with GDH, GS, and GOGAT serving as vital enzymes in NH_4_^+^ assimilation and NH_4_^+^ detoxification (Tabuchi et al., 2007). Our study found that *G. uralensis* supplied with low concentration NH_4_^+^ showed increased GDH, GS, and GOGAT gene expression. Interestingly, the expression level of NRTs, a set of DEGs acting as nitrate transporters responsible for nitrate uptake and regulation of nitrate-related genes (Zhu et al., 2019), was upregulated under low-level NH_4_^+^ condition but downregulated under NO_3_^-^ supply. This trend was similar to the gene expression changes observed in AMTs.

It has been reported that *α*-KG in the TCA cycle provides the carbon skeleton for both glutamate and glutamine. Over-assimilation of NH_4_^+^ increases organic acids such as *α*-KG and citrate in the TCA cycle, and this depletion of organic acids may result in altered growth (Zhang et al., 2017). Additionally, the GAD and SSADH genes, and the related metabolite GABA, were significantly enhanced in the control group. This indicated that nitrogen deficiency might stimulate the accumulation of GABA in *G. uralensis*. Pyruvate exists at the intersection of many biochemical pathways and is usually one of the final products of glycolysis (Baud et al., 2007). We observed significantly higher G6P, F6P, 3PGA, and PEP concentrations in the medium NH_4_^+^ treatment group. Simultaneously, PFK, PK, and PDH gene levels were upregulated both in low NH_4_^+^ and medium NO_3_^-^ treatments. Our results suggested that cultivating *G. uralensis* seedlings with appropriate concentrations of NH_4_^+^ or medium NO_3_^-^ can promote the synthesis of amino acids, the TCA cycle, and the glycolysis metabolism process.

Transcription factors are cis-acting elements that bind specifically to the DNA promoter region. They execute their roles by activating or inhibiting downstream target gene transcription (Deng et al., 2022). When *G. uralensis* is treated with different nitrogen, it involved 18 transcription factor families, including MYB, bHLH, WRKY, bZIP, etc. MYB TFs represent one of the most abundant transcription factor families in plants and exert transcriptional regulation effects on the biosynthesis of various secondary metabolites (Bai et al., 2021). GlyMYB genes in *G. uralensis* may primarily participate in hormone and stress responses, and are associated with flavonoid synthesis (Wang et al., 2021). Additionally, functional analysis of *G. uralensis* gene indicated that MYB and bHLH might regulate the biosynthesis of glycyrrhizin (Yang et al., 2022). As reported, GubHLH3 regulates soyasaponin biosynthetic genes and enhances the content of soyasapogenol B and sophoradiol in *G. uralensis* (Tamura et al., 2018). WRKY TFs are essential in plant growth and development, secondary metabolism, and abiotic and biotic stress response. They can combine with W-box TTGAC (C/T) in the target gene promoter to regulate the expression of downstream genes (Bai et al., 2021), and play a critical role in *G. uralensis’s* response to salt stress and drought stress (Goyal et al., 2020). Furthermore, bZIP TFs are implicated in plant responses to abiotic stress and secondary metabolite synthesis. Previous research has shown that bZIP TFs in *G. uralensis* might contribute to regulating the ABA signaling pathway (Han et al., 2021). This study suggested that the MYB, bHLH, WRKY, and bZIP transcription factors could be connected to regulating secondary metabolites in *G. uralensis* when exposed to various forms of nitrogen.

Furthermore, flavonoids and triterpenoids serve as crucial indicators of *G. uralensis* quality. Flavonoids, including flavones, flavonols, flavanones, anthocyanins, and isoflavones, these polyphenolic compounds possess properties such as reactive oxygen species scavenging, underlining their importance under abiotic stress in plants. (Guo et al., 2020; Zhang et al., 2022). Glycyrrhizic acid stands out as the characteristic bioactive triterpenic saponin in *G. uralensis* (Rizzato et al., 2017). However, our understanding of the influence mechanism of different nitrogen forms on flavonoids and glycyrrhizic acid in *G. uralensis* remains fragmented. In this study, we identified DEGs involved in forming chemical scaffolds for subsequent modifications in flavonoid synthesis. We observed an increase in specific flavonoid accumulations, paralleling changes in gene expression in *G. uralensis* in response to NH_4_^+^ or NO_3_^-^ conditions. Enzymes PAL, C4H, and 4CL play significant roles early in the flavonoid biosynthesis pathway. Phenylpropanoid biosynthesis begins with the PAL gene, which converts L-phenylalanine into *trans*-cinnamic acid, and plays a vital role in plant development and resistance to abiotic stresses in plants (Wang et al., 2021). CHR, CHI, F3H, and FLS are essential branch-point genes that regulate flavonoid accumulation (Park et al., 2019). The expression levels of PAL, C4H, 4CL, CHS, CHI, F3H and FLS positively correlated with the concentrations of glycyrrhizin, naringenin, and apigenin in the group treated with low concentration NH_4_^+^. Additionally, enzyme genes such as PAL, CHR, and CHI saw increased expression, aligning with the downstream products of isoliquiritigenin, liquiritigenin, and liquiritin in the low-concentration NO_3_^-^ treatment group. F3H affects the composition of dihydroxylated and trihydroxylated flavonoids, such as quercetin and kaempferol, which primarily scavenge reactive oxygen species and detoxify free radicals, thereby improving plant tolerance to environmental changes (Ulusoy and Sanlier, 2020). Notably, flavonoid downstream metabolites such as kaempferol, quercetin, and rutin increased markedly in the control group, suggesting that the absence of nitrogen treatment could trigger flavonoid accumulation. Moreover, glycyrrhizic acid synthesis begins with the MVA pathway of the terpene backbone. We discovered that the gene expression of structural triterpenoids, including HMGS, HMGR, SQS, and *β*-AS, increased substantially in the control, low ammonium nitrogen, and medium nitrate nitrogen groups. Conversely, the glycyrrhizic acid content exhibited varied increases under different nitrogen form treatments. This variation could arise from downstream modifications of glycyrrhizic acid, such as the oxidative reactions catalyzed by CYP 450 enzymes (Wang et al., 2021). Overall, low-level NH_4_^+^ or medium-level NO_3_^-^ treatment enhanced primary metabolism, covering amino acids, the TCA cycle, and glycolysis metabolism. Both low-level NH_4_^+^ and NO_3_^-^ treatment positively influenced secondary metabolism, especially flavonoid biosynthesis in *G. uralensis*. In essence, this research sheds light on how changes in nitrogen forms may improve the quality of *G. uralensis*. Future research can leverage these findings to develop improved cultivation strategies for this important medicinal plant.

## 5 Conclusion

In conclusion, this study delineates the physiological and global alterations in transcripts and metabolites inherent in *G. uralensis* when responding to different nitrogen forms. Medium-level ammonium nitrogen was found to significantly enhance *G. uralensis* root growth and biomass accumulation compared to nitrate nitrogen. By amalgamating transcriptomic and metabolic profiles, we have unveiled a broad stimulation of molecular and physiological metabolism of *G. uralensis* under individual NH_4_^+^ or NO_3_^-^ conditions. This encompassed improvements in nitrogen absorption and assimilation, glycolysis, TCA cycle, as well as flavonoid and triterpenoid metabolism. This research deepens our understanding of the intricate relationships between responsive genes and the ensuing metabolic reactions. It also lays the groundwork for a holistic and integrated analysis of molecular responses to distinct nitrogen forms in *G. uralensis*. Our findings also provide invaluable data for delving into the molecular and chemical mechanisms that drive the flavonoid biosynthesis pathway in *Glycyrrhiza* plants when exposed to various nitrogen forms.

## Data availability statement

The original contributions presented in the study are publicly available. This data can be found here: https://www.ncbi.nlm.nih.gov/bioproject/PRJNA970183.

## Author contributions

ZS conceived the study and processed the experimental data. YC, YZ, ZZ, YJ, SW, and YW cultivated samples and conducted the experiments. YC and YB performed the RNA extraction, the qRT-PCR experiments, and data analysis. YC wrote the manuscript. All authors contributed to the article and approved the submitted version.

## Glossary

**Table:**

KEGG	Kyoto encyclopedia of genes and genomes
GO	Gene ontology
DEGs	Differentially expressed genes
VIP	Variable importance of the projection
OPLS-DA	Orthogonal partial least squares discriminant analysis
DAMs	Differentially accumulated metabolites
FPKM	Fragments per kilobase of transcript per million fragments mapped
FDR	False discovery rate
LIT	Linear ion trap
qRT-PCR	Quantitative real-time polymerase chain reaction
CC	Cell component
MF	Molecular function
BP	Biological process
TFs	Transcription factors
LC-ESI-MS/MS	Liquid chromatography-electrospray ionization-tandem mass spectrometry
NRT	Nitrate transporters
NR	Nitrate reductase
NiR	Nitrite reductase
GS	Glutamine synthetase
GOGAT	Glutamate synthetase
GDH	Glutamate dehydrogenase
AMTs	Ammonium transporters
Gln	Glutamine
Glu	Glutamate
*α*-KG	*α*-ketoglutarate
TCA cycle	Tricarboxylic acid cycle
OAA	Oxaloacetate
KGDH	*α*-ketoglutarate dehydrogenase
CL	Citrate synthase
IDH	Isocitric dehydrogenase
GABA	*γ*-Aminobutyric acid
GAD	Glutamate decarboxylase
GABA-T	GABA transaminase
SUCS	Succinic semi-aldehyde
SSADH	Succinic semi-aldehyde dehydrogenase
PK	Pyruvate kinase
PFK	Phosphofructokinase
PDH	Phosphofructokinase
3PGA	3-phosphoglyceric acid
G6P	Glucose-6-phosphate
F6P	Fructose-6-phosphate
PEP	Phosphoenolpyruvic acid
HK	Hexokinase
FUM	Fumarase
MDH	Malate dehydrogenase
AspAT	Aspartate aminotransferase
Asp	Aspartic acid
MVA	Mevalonic acid
PAL	Phenylalanine ammonia-lyase
C4H	Trans-cinnamate 4-monooxygenase
4CL	4-coumarate-CoA ligase
CHS	Chalcone synthase
CHR	Chalcone reductase
CHI	Chalcone isomerase
F3H	Flavanone 3- hydroxylase
FLS	Flavonol synthase
F3′H	Flavonoid 3′-hydroxylase
GT	Glycosyltransferases
PDT	Prephenic acid dehydratase
AAT	Aromatic amino acid aminotransferase
ACC	Acetyl CoA carboxylase
ACAT	Acetyl CoA C-acetyltransferase
HMGS	Hydroxymethylglutaryl-CoA synthase
HMGR	3-hydroxy-3-methylglutaryl-CoA reductase
MK	Mevalonate kinase
MPK	Mevalonate phosphate kinase
MPD	Mevalonate pyrophosphate decarboxylase
IDI	Isopentenyl diphosphate isomerase
FPPS	Farnesyl pyrophosphate synthase
SQS	Squalene synthase
*β*-AS	*β*-amyrin synthase.
