# Supplementary material for: Transcriptomics and metabolomics reveal the primary and secondary metabolism changes in Glycyrrhiza uralensis with different forms of nitrogen utilization
Source: Front Plant Sci. 2023 Nov 2;14:1229253. doi: 10.3389/fpls.2023.1229253 (PMC10653330; doi:10.3389/fpls.2023.1229253)
Supplement: Supplementary file 2 [file DataSheet_1.docx]

**Transcriptomics and metabolomics reveal the primary and secondary metabolism changes in *Glycyrrhiza uralensis* with different forms of nitrogen utilization**

**
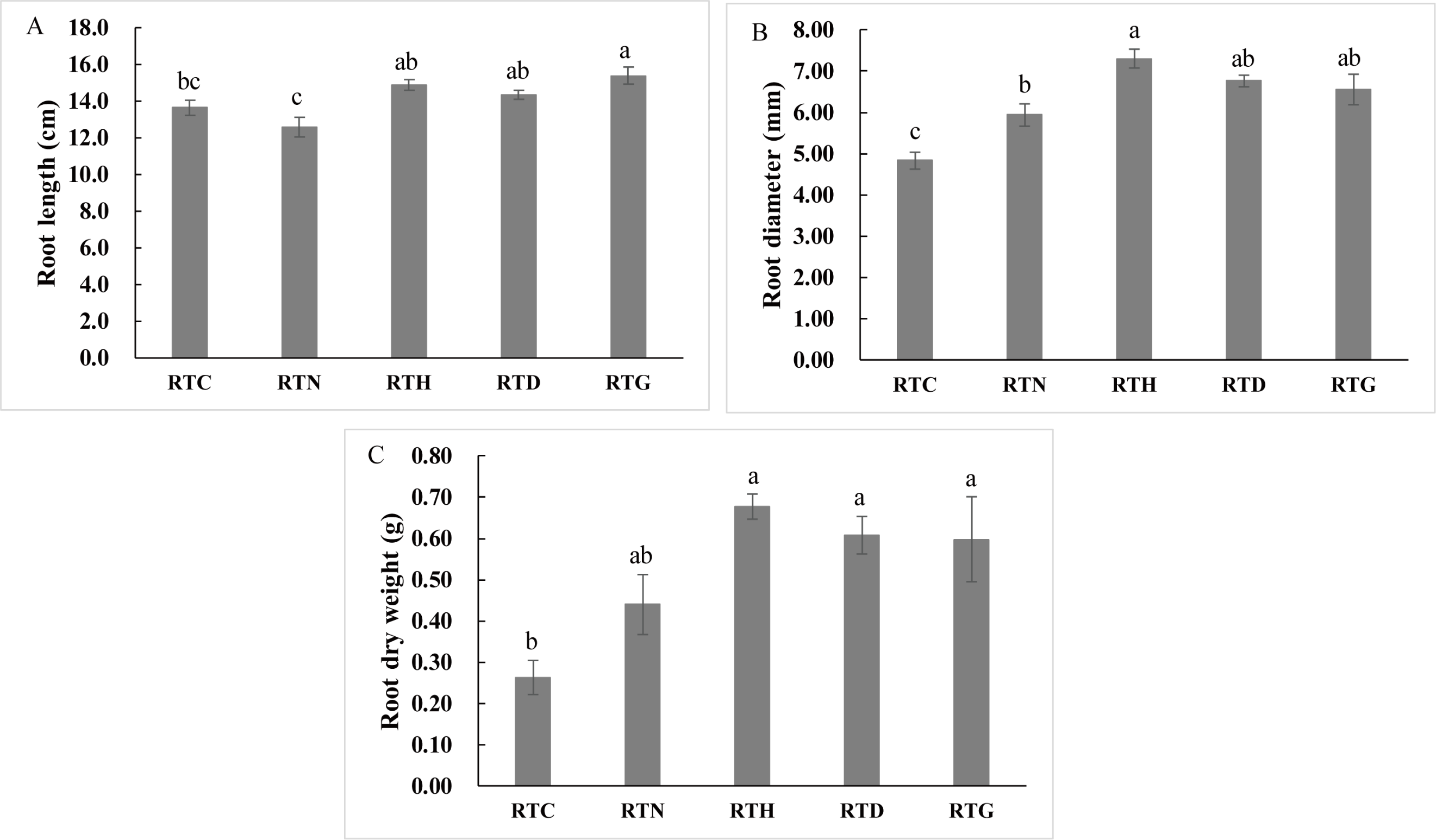
**

Figure S1 The biomass of *G. uralensis* under different nitrogen treatment

(A) Root length, (B) Root diameter, (C) Root dry weight. Different lowercase letters for the same indicator indicated significant differences between groups （*P* < 0.05）.


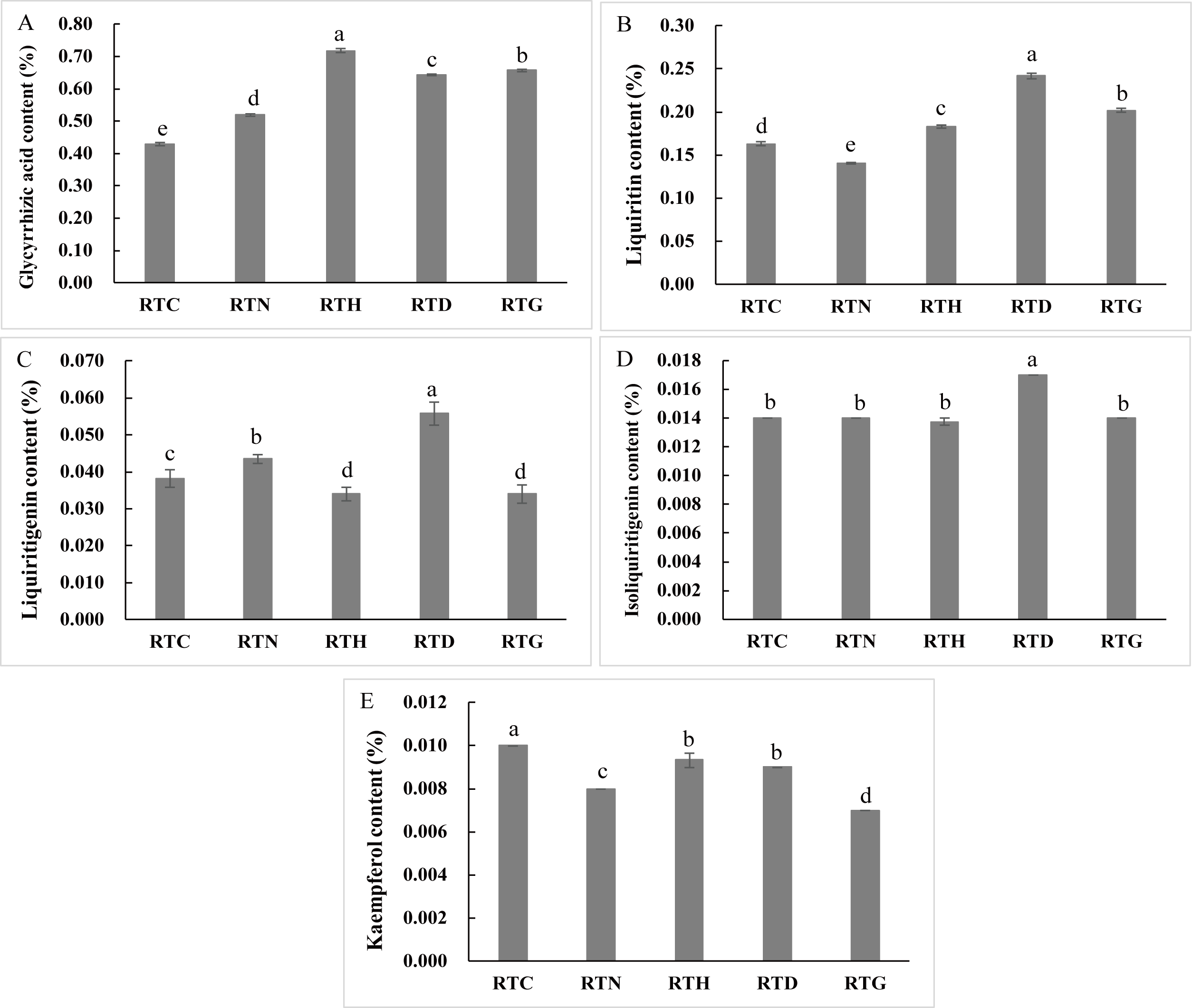


Figure S2 The main active ingredients content of *G. uralensis* under different nitrogen treatment

(A) Glycyrrhizic acid content, (B) Liquiritin content, (C) Liquiritigenin content, (D) Isoliquiritigenin content, (E) Kaempferol content. Different lowercase letters for the same indicator indicated significant differences between groups （*P* < 0.05）.


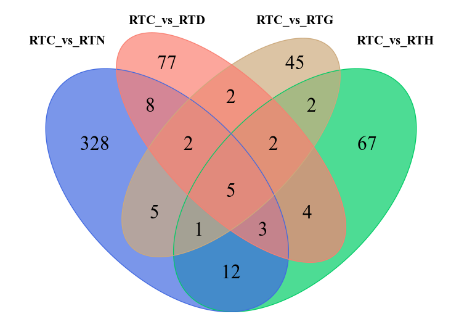


Figure S3 Venn diagram of differentially expressed genes (DEGs).


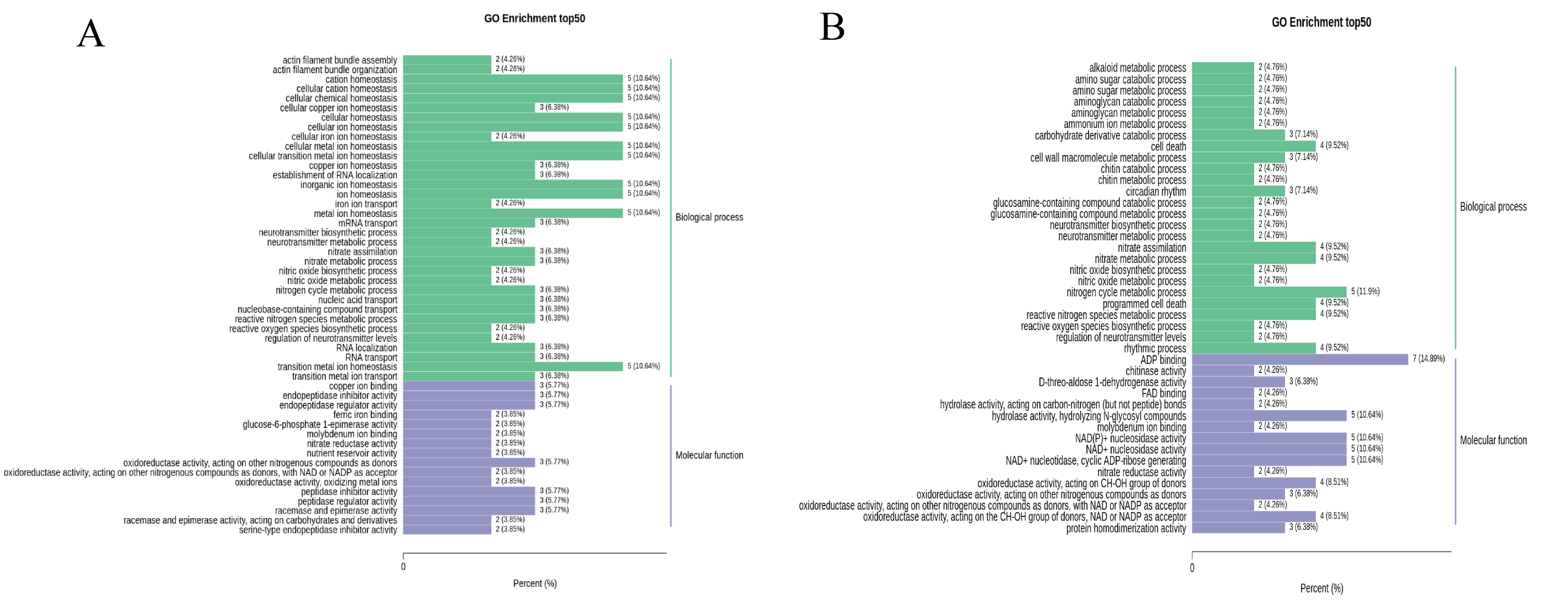


Figure S4 Gene ontology (GO) functional classifications of differentially expressed genes (DEGs) of RTC_vs_RTD (A) and RTC_vs_RTG (B).


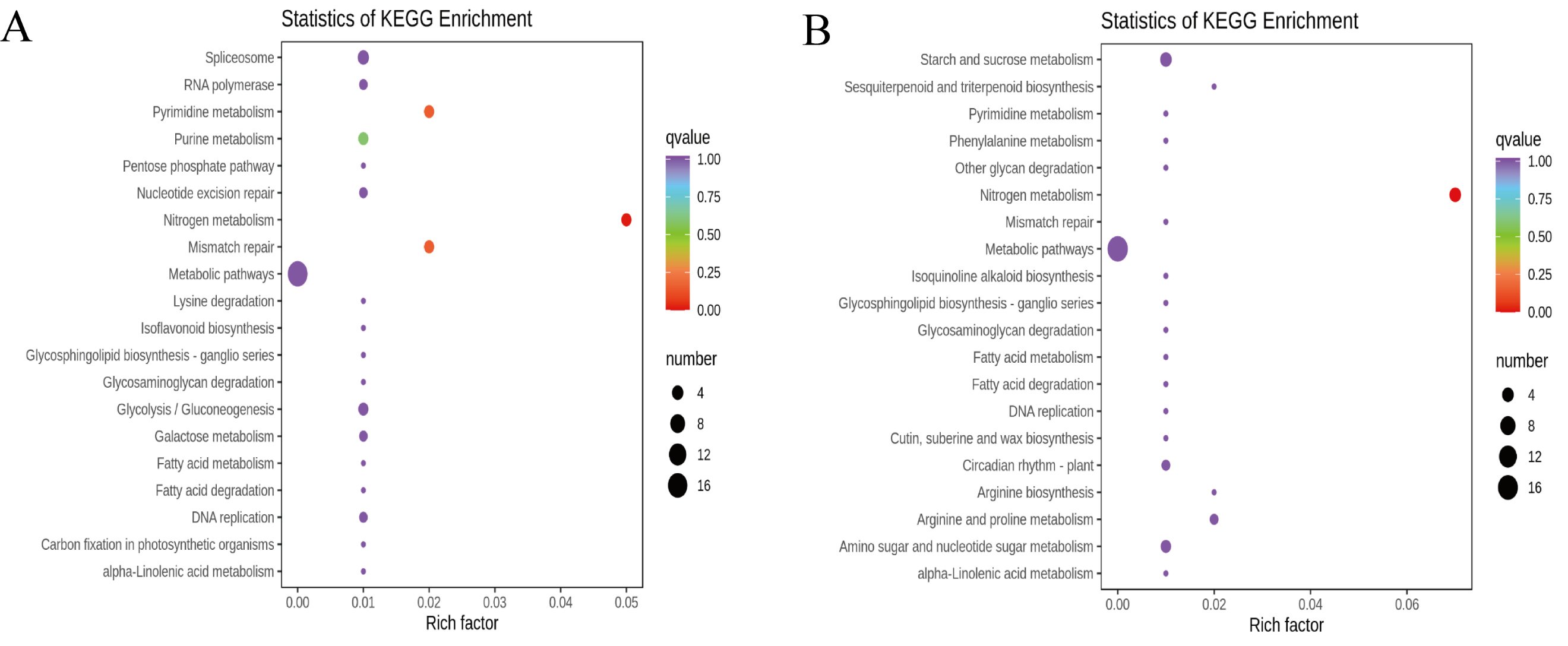


Figure S5 DEGs KEGG enrichment of top 20 pathways in RTC_vs_RTD (A) and RTC_vs_RTG (B).


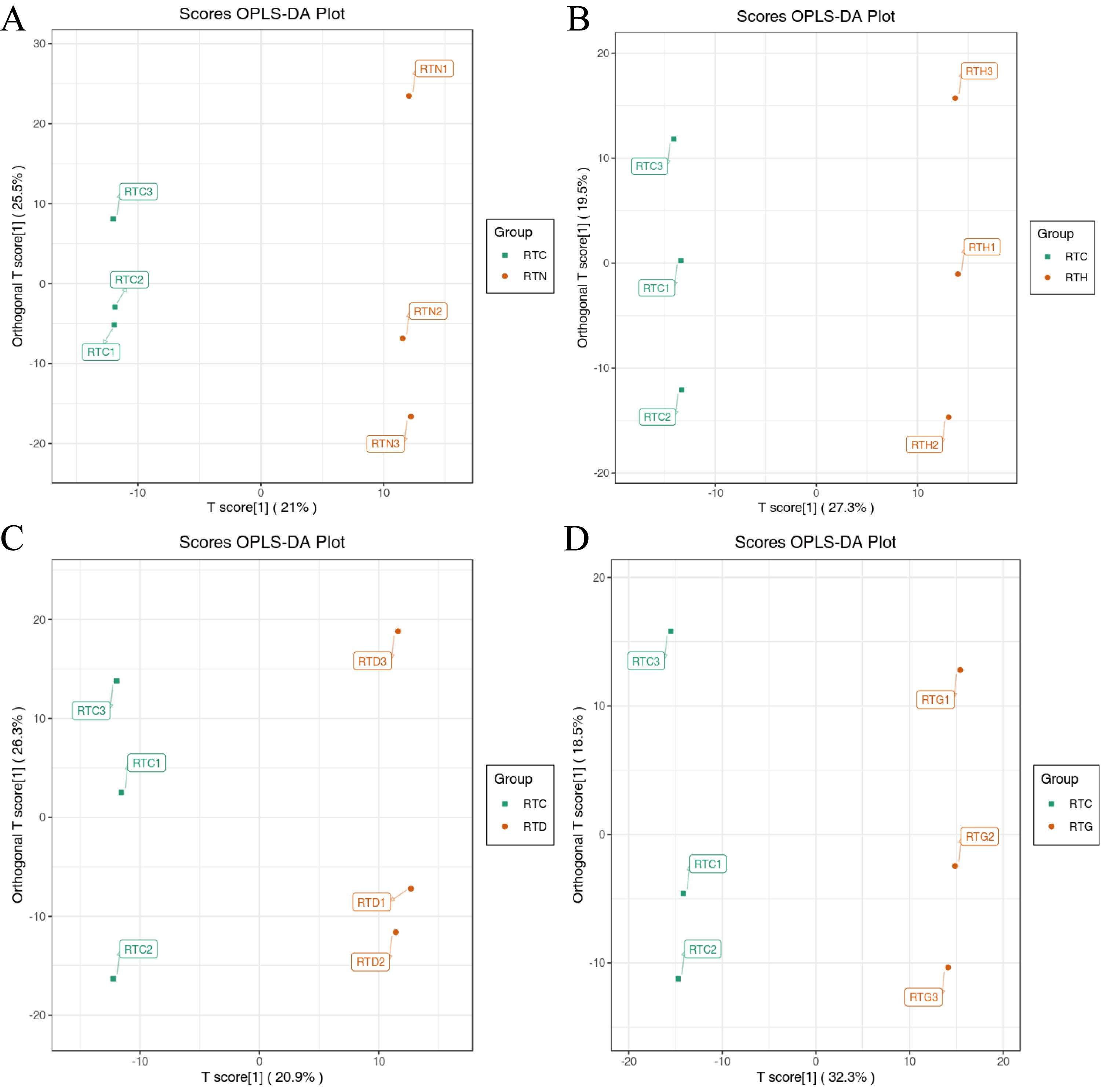


Figure S6 Metabolics analysis of different forms of nitrogen treatment *G. uralensis* samples using an OPLS-DA analysis. (A) RTC_vs_RTN, (B) RTC_vs_RTH, (C) RTC_vs_RTD, (D) RTC_vs_

RTG.


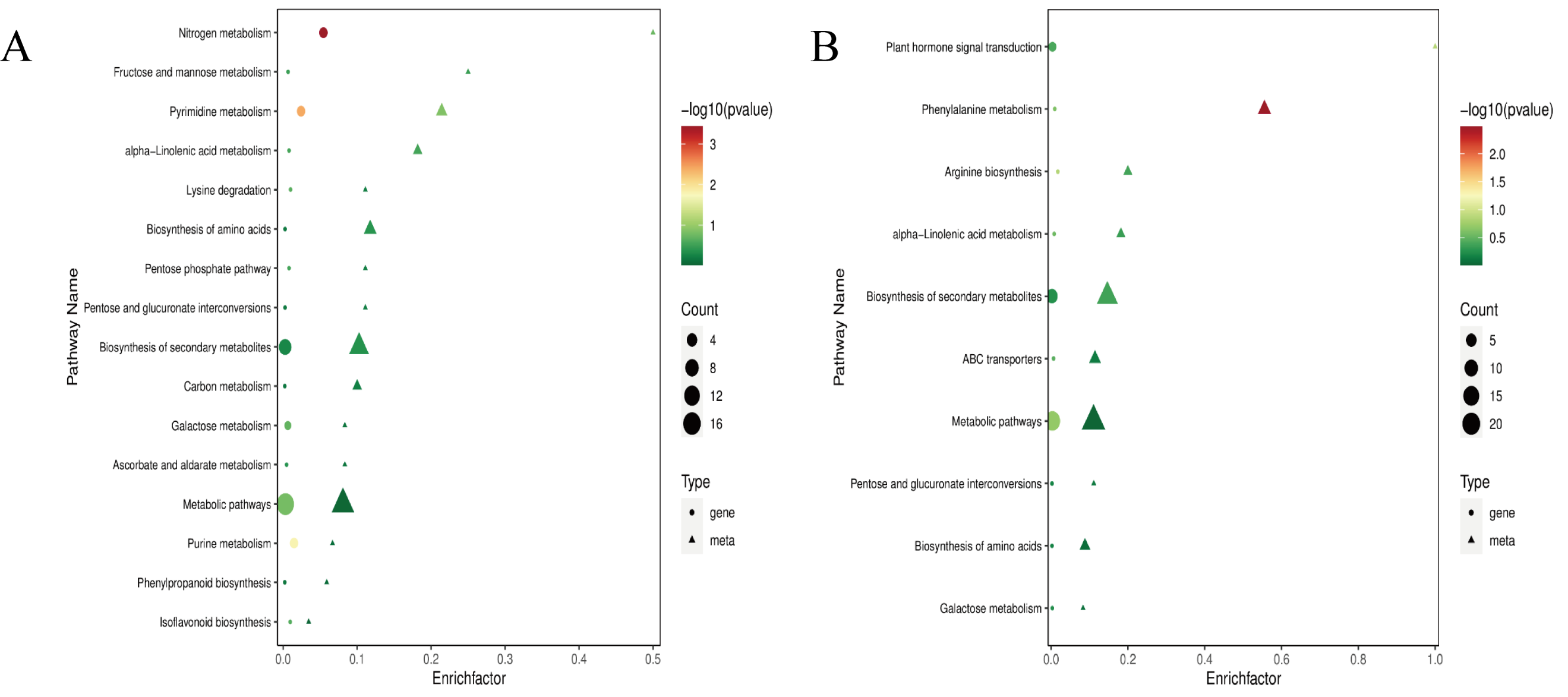


Figure S7 Joint pathways of DEGs and DAMs KEGG enrichment in RTC_vs_RTD (A) and RTC_vs_RTG (B).


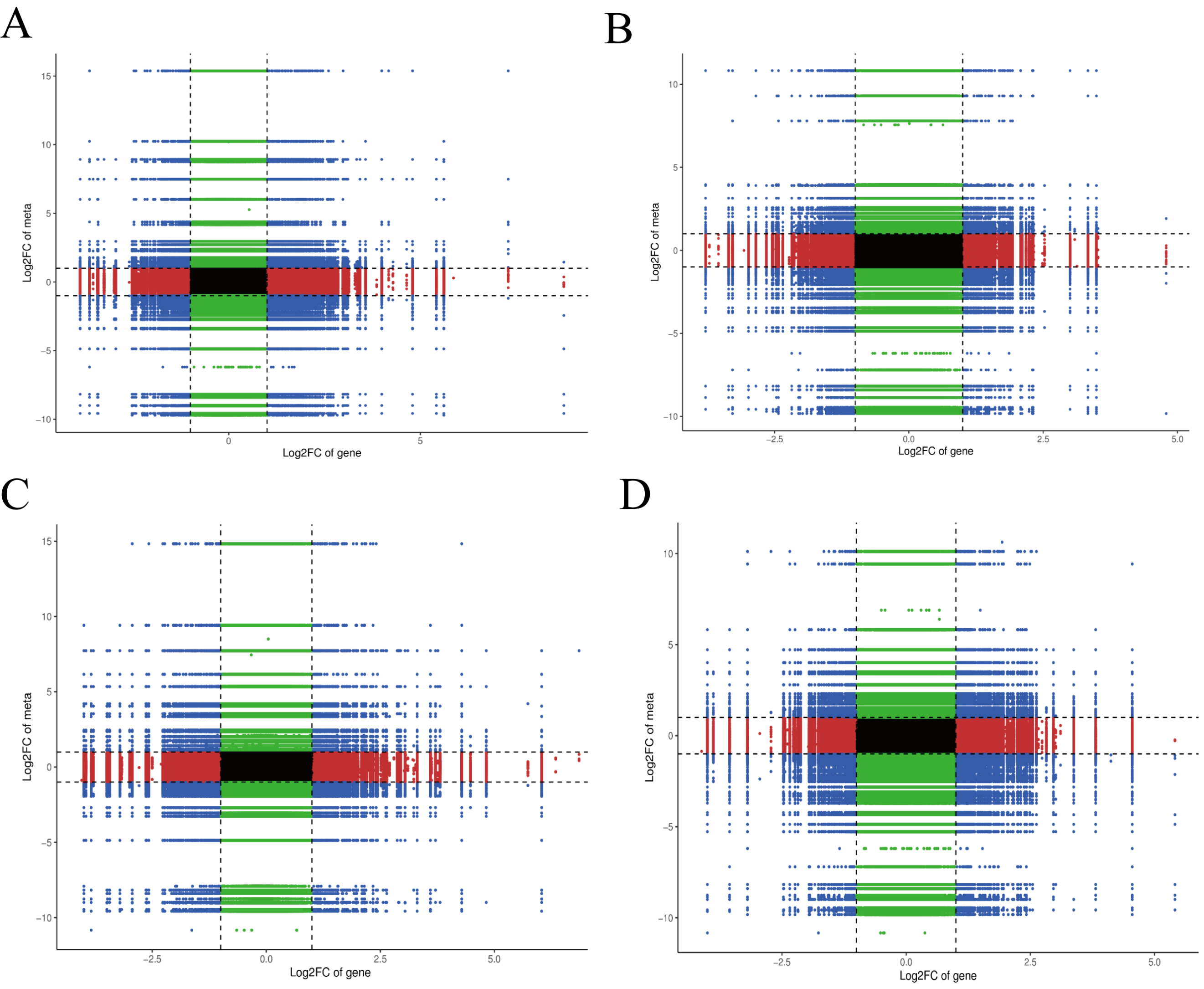


Figure S8 Quadrant diagram representing the association of transcriptomics and metabolomics variations in RTC_vs_RTN (A), RTC_vs_RTH (B), RTC_vs_RTD (C), and RTC_vs_RTG (D).

The black dotted lines represent the differential thresholds. Outside the threshold lines, there are significant differences in the gene/metabolites, whereas within the threshold lines the gene/metabolites are unchanged. Each point represents a gene/metabolite. Black dots = unchanged genes/metabolites, green dots = differentially accumulated metabolites with unchanged genes, red dots = differentially expressed genes with unchanged metabolites, and blue dots = both differentially expressed genes and differentially accumulated metabolites.
